# Nanoparticle-Mediated Targeting of Cyclosporine A Enhances Cardioprotection Against Ischemia-Reperfusion Injury Through Inhibition of Mitochondrial Permeability Transition Pore Opening

**DOI:** 10.1038/srep20467

**Published:** 2016-02-10

**Authors:** Gentaro Ikeda, Tetsuya Matoba, Yasuhiro Nakano, Kazuhiro Nagaoka, Ayako Ishikita, Kaku Nakano, Daiki Funamoto, Kenji Sunagawa, Kensuke Egashira

**Affiliations:** 1 Department of Cardiovascular Medicine, Fukuoka, Japan; 2 Department of Cardiovascular Research, Development, and Translational Medicine, Kyushu University Graduate School of Medical Sciences, Fukuoka, Japan

## Abstract

Myocardial ischemia-reperfusion (IR) injury limits the therapeutic effects of early reperfusion therapy for acute myocardial infarction (MI), in which mitochondrial permeability transition pore (mPTP) opening plays a critical role. Our aim was to determine whether poly-lactic/glycolic acid (PLGA) nanoparticle-mediated mitochondrial targeting of a molecule that inhibits mPTP opening, cyclosporine A (CsA), enhances CsA-induced cardioprotection. In an *in vivo* murine IR model, intravenously injected PLGA nanoparticles were located at the IR myocardium mitochondria. Treatment with nanoparticles incorporated with CsA (CsA-NP) at the onset of reperfusion enhanced cardioprotection against IR injury by CsA alone (as indicated by the reduced MI size at a lower CsA concentration) through the inhibition of mPTP opening. Left ventricular remodeling was ameliorated 28 days after IR, but the treatment did not affect inflammatory monocyte recruitment to the IR heart. In cultured rat cardiomyocytes *in vitro*, mitochondrial PLGA nanoparticle-targeting was observed after the addition of hydrogen peroxide, which represents oxidative stress during IR, and was prevented by CsA. CsA-NP can be developed as an effective mPTP opening inhibitor and may protect organs from IR injury.

Innovative therapeutic strategies for protecting the heart from ischemia-reperfusion (IR) injury are necessary to adequately reduce infarct size and improve clinical outcomes in acute myocardial infarction (MI) patients who undergo reperfusion therapy^1^,^2^,^3^. Accumulating evidence suggests that the opening of the mitochondrial permeability transition pore (mPTP), which is a non-selective, high-conductance channel located in the inner mitochondrial membrane, mediates necrosis/apoptosis in the early phase of IR injury. The mPTP remains closed during ischemia and only opens in the first few minutes after myocardial reperfusion in response to mitochondrial oxidative stress^1^. mPTP opening induces mitochondrial membrane potential collapse, cytochrome c efflux, ATP breakdown, and ultimately, cardiomyocyte death. Therefore, mPTP is a critical mediator of cardiomyocyte death in the early phase of IR injury. Genetic ablation of cyclophilin D, which is a key regulatory molecule for mPTP opening, markedly reduces IR injury in mice^4^,^5^. In addition, intravenous administration of an mPTP-opening inhibitor, cyclosporine A (CsA), at the time of reperfusion can reduce myocardial IR injury in animals and patients with acute MI^6^. However, not all studies have reported cardioprotective effects from CsA (Supplemental Table 1). Piot *et al*.^6^ conducted an important clinical trial in 58 patients who had an ST-elevation acute MI and reported that an intravenous bolus administration of 2.5 mg/kg CsA at the time of reperfusion therapy reduced the MI size by 20%, which was assessed by creatinine kinase and troponin I release; however, the benefit from CsA was insufficient to prevent cardiac remodeling^7^. In a phase 3 CIRCUS (Dos Cyclosporine Improve Clinical Outcome in STEMI Patients) study conducted by the same group, in patients with anterior ST-elevation acute MI who had been referred for primary percutaneous coronary intervention, intravenous cyclosporine at a dose of 2.5 mg/kg (395 patients) did not result in better clinical outcomes than those with placebo (396 patients) and did not prevent adverse left ventricular remodeling at 1 year^8^. The inconclusive efficacy of intravenous CsA in experimental and clinical studies raises the possibility of an insufficient local drug concentration at the therapeutic CsA target when it is administered at the time of reperfusion. Therefore, from a clinical perspective, it is essential to develop a drug delivery system that facilitates CsA delivery to the target IR injury sites (mitochondrion) during reperfusion.

Recently, we developed a nanoparticle-mediated drug delivery system that uses bioabsorbable poly-lactic/glycolic acid (PLGA) nanoparticles^9^,^10^,^11^,^12^,^13^,^14^,^15^. Nano-size materials accumulate in injured tissues, including the IR myocardium, where vascular permeability is enhanced^16^,^17^; the materials were rapidly taken up by circulating monocytes and reticuloendothelial phagocytic organs after intravenous administration^18^,^19^. Thus, PLGA nanoparticles are a clinically feasible drug delivery system for an IR injury. However, it is unknown whether the use of nanoparticles to target mitochondria-acting drugs, such as CsA, protects the heart from IR injury. In the present study, we engineered PLGA nanoparticles that incorporate CsA (CsA-NP). We traced PLGA nanoparticles that were intravenously injected at the time of reperfusion and demonstrated CsA delivery to the IR myocardium, as well as its mitochondria. We then tested the hypothesis that nanoparticle-mediated CsA targeting to the IR myocardium mitochondria enhances CsA-induced cardioprotection through the inhibition of mPTP opening.

## Results

### *In vivo* mitochondria-targeting PLGA nanoparticle properties in the IR myocardium

**Figure 1:**
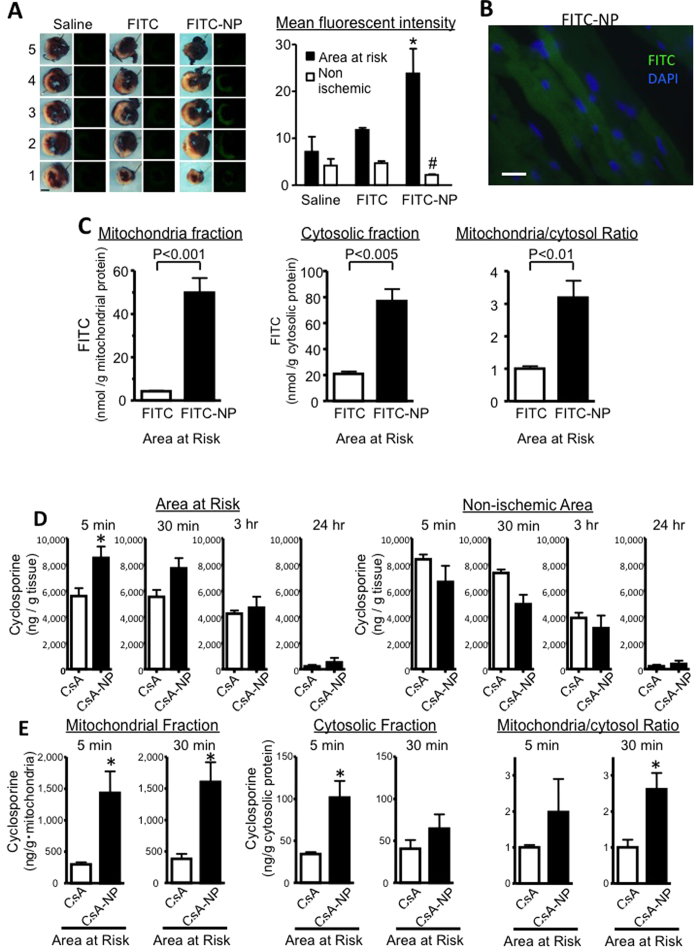
Mitochondria-targeting properties of PLGA nanoparticles in myocardial IR injury *in vivo*. (**A**) Representative light (left) and fluorescence (right) photographs of the heart cross-sections (the number of cross-sections are indicated from the apex to the base) 3 h after an intravenous injection of saline, FITC alone, or FITC-NP. In the light images, the hearts were stained with Evans blue and TTC to determine the area at risk. Scale bar: 1 mm. The right bar graph indicates the mean fluorescence intensity in the area at risk and the non-ischemic myocardium of the 3^rd^ axial cross section from the apex. The data represent the mean ± SEM (N = 3 per bar). *P < 0.05 versus ischemic myocardium in the area at risk, and ^#^P < 0.05 versus saline according to a two-way ANOVA followed by Bonferroni’s multiple comparison test. (**B**) Fluorescence microscopy images for the cross-sections in the infarct border area obtained from FITC-NP-treated animals. The nuclei are identified by DAPI staining (blue). Scale bar: 20 μm. (**C**) The levels of FITC in the mitochondrial and cytosolic fractions from the excised ischemic myocardium 5 min after reperfusion in animals that were treated with FITC alone or FITC-NP. The data represent the mean ± SEM (N = 4 each) and were analyzed with using unpaired *t*-tests. (**D**) The CsA concentrations in IR myocardial and non-ischemic myocardial tssue after an intravenous injection of 2.5 mg/kg CsA or CsA-NP that contained 2.5 mg/kg CsA. The data represent the mean ± SEM (N = 6 each). *P < 0.05 versus non-ischemic myocardial tissues according to unpaired *t*-tests. (**E**) CsA concentrations in the mitochondrial fractions 5 and 30 min after treatment. The data represent the mean ± SEM (N = 4 each). *P < 0.05 versus CsA treated groups according to unpaired *t*-tests.

We examined the distribution of FITC in the heart 3 h after an intravenous injection of FITC-NP at the time of reperfusion. FITC-NP was distributed in the infarct area and the area at risk; however, no FITC signals were observed after treatment with the same quantity of FITC alone or with the saline (Fig. 1A). The fluorescence microscopy images were magnified and exhibited FITC signals from cardiomyocytes with striations in the infarct border zone (Fig. 1B). Transmission electron microscopy analysis in mouse hearts 30 min after IR revealed PLGA nanoparticles localized in the small vessel lumen and the immediate vicinity of the mitochondria in IR-injured myocardium cardiomyocytes (Supplemental Fig. 1). In contrast, no nanoparticle-like structure was observed in the non-ischemic myocardium.

We subsequently isolated mitochondria and cytosolic fractions from the ischemic myocardium 5 min after IR, measured the quantity of FITC per protein. FITC-NP was delivered to the IR myocardium mitochondria and cytosol (Fig. 1C). The Mitochondria/Cytosol Ratio was greater in FITC-NP group than in FITC solution group.

We examined CsA pharmacokinetics in the IR mouse model that was intravenously treated with a CsA solution (2.5 mg/kg) or CsA-NP (contained 2.5 mg/kg CsA) at the time of reperfusion. CsA-NP increased the IR myocardium CsA concentration (the area at risk) only during the early phase (5 min) after reperfusion, but not at later time points; however, we did not observe selective CsA delivery in the non-ischemic myocardium (Fig. 1D). In contrast, the CsA-NP yielded an approximately 5-fold higher CsA concentration in the mitochondrial fractions from the IR myocardium 5 and 30 min after reperfusion (Fig. 1E). CsA-NP yielded higher CsA concentrations compared with a CsA solution in the reticuloendothelial phagocytic organs (i.e., spleen, liver, lung and whole blood) (Supplemental Table 2). An *in vitro* analysis of cyclosporine release kinetics showed an early burst of cyclosporine release, in which approximately 50% of the total amount was released within 6 hours (Supplementary Fig. 2). These findings suggest CsA-NP was preferentially delivered to the IR myocardium mitochondria and cytosol in the area at risk in this model.

### CsA-NP conferred superior cardioprotection against myocardial IR injury from CsA alone through the blockage of mPTP opening

**Figure 2:**
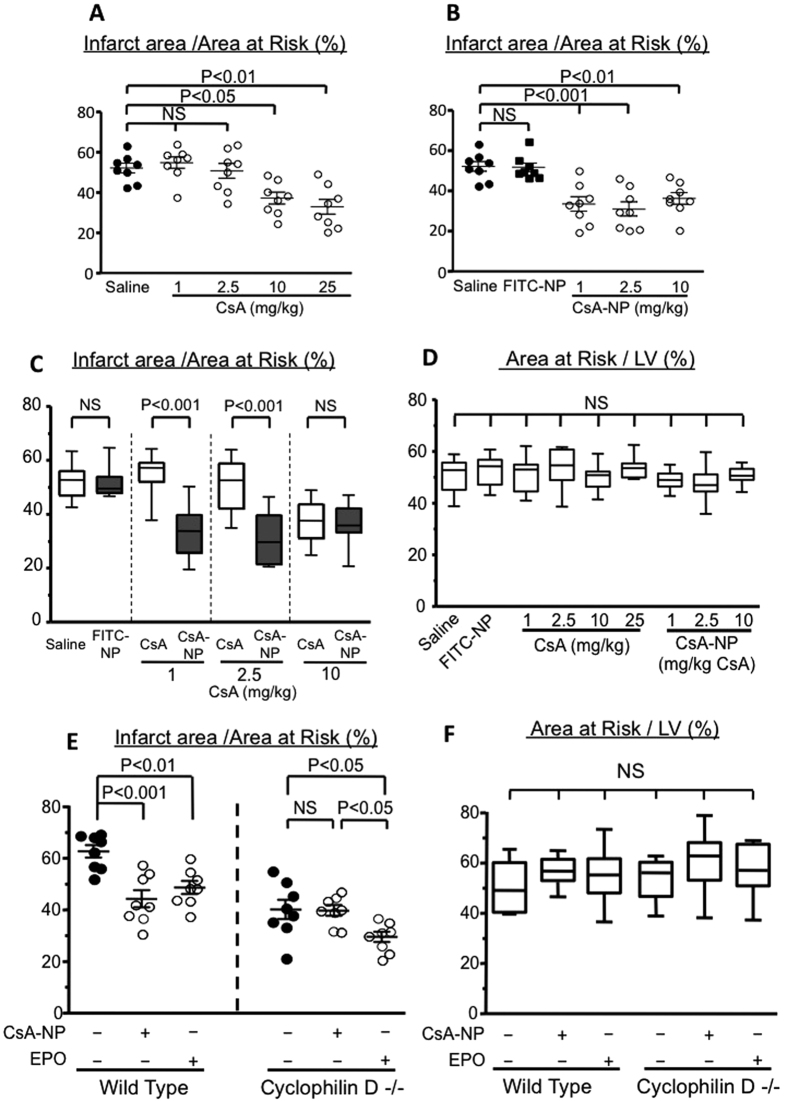
The effects of CsA and CsA-NP on IR injury (MI Size) in mice. (**A**) CsA effects on MI size. The data represent the mean ± SEM (N = 8 each). (**B**) CsA-NP effects on MI size. The data represent the mean ± SEM (N = 8 each) and were compared using a one-way ANOVA followed by Bonferroni’s multiple comparison test. (**C**) A comparison of the effects of CsA and CsA-NP on MI size. (**D**) The area at risk as a percentage of the left ventricle (LV). Each bar represents the mean ± SEM (N = 8 each), and the data were compared using a one-way ANOVA followed by Bonferroni’s multiple comparison test. (**E**) The effects of CsA-NP and erythropoietin (EPO) administration on the MI size in wild-type and cyclophilin D^−/−^ mice. The data represent the mean ± SEM (N = 8). (**F)** The area at risk as a percentage of the left ventricle. Each bar represents the mean ± SEM (N = 8 each). The data were compared using a one-way ANOVA followed by Bonferroni’s multiple comparison test.

Intravenous CsA treatment at the onset of reperfusion reduced IR injury (MI size) in a dose-dependent manner (Fig. 2A), and higher doses of CsA (10 and 25 mg/kg) yielded therapeutic effects. In contrast, CsA-NP, which contained 1.0 mg/kg CsA, reduced the MI size; however, we did not observe a further reduction in the MI size following treatment with CsA-NP that contained higher doses of CsA (2.5 and 10 mg/kg; Fig. 2B). FITC-NP was used as a control and did not exhibit therapeutic effects. Treatment with CsA-NP was at least 25-fold more effective at reducing IR injury compared with CsA treatment alone (Fig. 2C). Additionally, the percentage of area at risk (AAR) in the left ventricle was comparable between all study groups (Fig. 2D).

We subsequently examined the role of mPTP opening in the CsA-NP-mediated cardioprotection mechanisms against IR injury. CsA inhibits mPTP opening through its action on a critical mPTP regulator, cyclophilin D. Therefore, we examined the therapeutic effects of CsA-NP that contained CsA at 1.0 mg/kg in cyclophilin D^−/−^ mice, which exhibited reduced myocardial IR injury to a similar extent as CsA-NP in WT mice (Fig. 2E,F). In the cyclophilin D^−/−^ mice, we did not observe the CsA-NP-mediated reduction in myocardial IR injury. In contrast, intravenous treatment with erythropoietin (2500 U/kg × 2) 24 h and 30 min prior to the induction of myocardial ischemia, which promotes cardioprotection from IR injury through cyclophilin D-independent mechanisms^20^, reduced IR injury in the cyclophilin D^−/−^ mice. Cardioprotection against IR injury by CsA-NP may be mediated, in part, through the inhibition of the calcium-activated protein phosphatase calcineurin because CsA inhibits calcineurin^21^. We measured calcineurin activity in the IR myocardium 3 h after an intravenous injection of saline, 1.0 mg/kg CsA, 10 mg/kg CsA, or CsA-NP that contained 1.0 mg/kg CsA and did not observed a difference in calcineurin activity between the study groups (Supplemental Table 3). These results suggest that the primary mechanism that underlies CsA-NP-induced cardioprotection against IR injury was mediated through the inhibition of mPTP opening.

**Figure 3:**
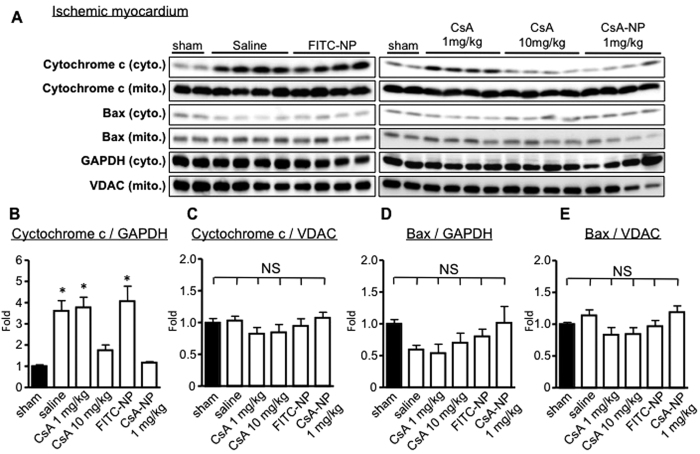
Effects of CsA and CsA-NP on cytochrome c leakage in the IR myocardium. (**A**) Mitochondrial and cytosolic fractions from ischemic myocardia were analyzed via immunoblotting for mitochondrial Bax recruitment and cytochrome c release into the cytosol in the sham, saline, FITC-NP, CsA at 1 mg/kg, CsA at 10 mg/kg, or CsA-NP that contained 10 mg/kg CsA groups. (**B–E**) Bar graphs indicating the normalized fold changes for cytochrome c leakage into the cytosol and Bax translocation to the mitochondria. The data represent the mean ± SEM (N = 8 each). *P < 0.001 versus the sham group based on a one-way ANOVA followed by Bonferroni’s multiple comparison test.

We examined cytochrome c leakage from the mitochondria into the cytosol 30 min after IR as a result of mPTP opening using western blotting of the cytosolic and mitochondrial fractions of the IR myocardium. Cytochrome c leakage was observed in the mice treated with saline, FITC-NP and 1.0 mg/kg CsA. In contrast, treatment with 10 mg/kg CsA or CsA-NP that contained 1.0 mg/kg CsA inhibited cytochrome c leakage (Fig. 3A,B). Bax is a critical pro-apoptotic protein, and Bax translocation from the cytosol to the mitochondria triggers apoptosis via mitochondrial outer membrane permeabilization, which induces cytochrome c leakage^4^,^5^. Bax translocation was not observed in the study groups, which suggests the observed cytochrome c leakage primarily depends on mPTP opening. Furthermore, cytochrome c leakage and Bax translocation were not observed in the non-ischemic myocardium (Supplemental Fig. 3).

These results suggested that the primary mechanism that underlies CsA-NP induced cardioprotection against IR injury was mediated through the inhibition of mPTP opening.

### CsA-NP ameliorated cardiac dysfunction and remodeling 4 weeks after IR

Echocardiography demonstrated CsA-NP that contained 1.0 mg/kg CsA, but not saline or 1.0 mg/kg CsA, ameliorated an enlargement of the left ventricular dimension, a decrease in the left ventricular ejection fraction (LVEF) and LV fractional shortening (LVFS) 4 weeks after IR (Supplemental Table 4). We did not observe differences in the blood pressure or heart rate between the study groups (Supplemental Table 5).

### CsA-NP ameliorated IR injury without impacting recruitment of inflammatory monocytes to the IR heart

**Figure 4:**
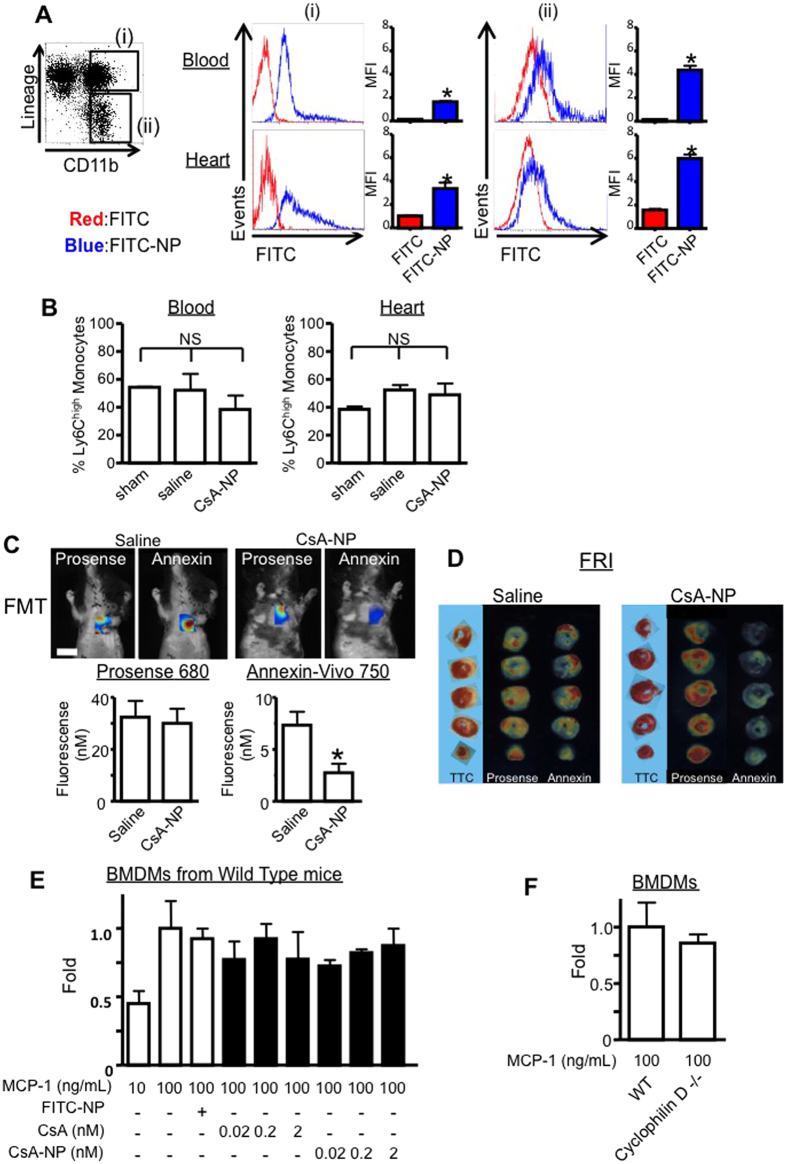
CsA-NP effects on inflammatory cells. (**A**) Histograms illustrate FITC-NP uptake in inflammatory cells. Scatter plot graphs illustrate FITC MFI quantification in inflammatory cells. The data represent the mean ± SEM (N = 4 per group). (**B**) The percentage of Ly6C^high^ monocytes in the peripheral blood and heart. (**C**) Coronal FMT image acquired after co-administration of Prosense-680 and Annexin-Vivo 750. Quantification of Prosense-680 and Annexin-Vivo 750 activation 24 h after IR. The data represent the mean ± SEM (N = 8 per group). The data were compared using a one-way ANOVA followed by Bonferroni’s multiple comparison test. Scale bar: 10 mm. FMT: fluorescence molecular tomography. (**D**) TTC stain and FRI imaging of the heart sections 24 h after IR. Scale bar: 1 mm. FRI: fluorescence reflectance imaging. (**E**,**F**) The effects of a CsA-NP or cyclophilin D deficiency on MCP-1-induced monocyte chemotaxis in BMDM. The bar graphs show the normalized fold changes. The data represent the mean ± SEM (N = 6 per group). The data were compared using a one-way ANOVA followed by Bonferroni’s multiple comparison test.

At 24 hours of reperfusion, flow cytometry analysis was performed to examine impact of recruitment of inflammatory monocytes to the IR heart. FITC-NP was taken up by leukocytes (CD11b ^+^/Lin^+^ neutrophil or CD11b^+^ /Lin^−^ monocytes) in the blood and myocardium from the mice treated with FITC-NP at the time of reperfusion (Fig. 4A). We next examined the role of Ly6C^high^ inflammatory monocytes 24 h after treatment with a saline solution or CsA-NP at the time of reperfusion through a flow cytometry analysis. We did not observe differences in the numbers of total monocytes or Ly6C^high^ inflammatory monocytes for the peripheral blood between the two groups (Fig. 4B). These data suggest CsA-NP do not affect inflammatory monocyte recruitment to the IR heart from the peripheral blood.

We also performed *in vivo* dual channel fluorescence molecular tomography (FMT) imaging and *ex vivo* fluorescence reflectance imaging (FRI) to visualize and simultaneously measure protease activity (inflammation) and cellular death in the IR heart after co-administration of sensors for pan-cathepsin protease (Prosense-680) and phosphatidylserine exposure (AnnexinVivo-750). Consistent with the flow cytometry analysis, CsA-NP did not exhibit effects on inflammatory protease activity. In contrast, CsA-NP reduced the level of cell death (Fig. 4C). FRI revealed AnnexinVivo signals localized in the infarct area demarcated by TTC staining, and prosense-680 signals located peri-infarct area (Fig. 4D).

We used bone marrow-derived macrophages (BMDMs) to examine chemotactic activity in response to MCP-1. CsA and CsA-NP did not exhibit inhibitory effects against chemotaxis in the BMDMs from the WT mice (Fig. 4E). In addition, the chemotactic activity did not differ between the BMDMs from the WT or cyclophilin D^−/−^ mice (Fig. 4F).

### Mitochondria-targeting PLGA nanoparticle properties were associated with mPTP opening *in vitro*

**Figure 5:**
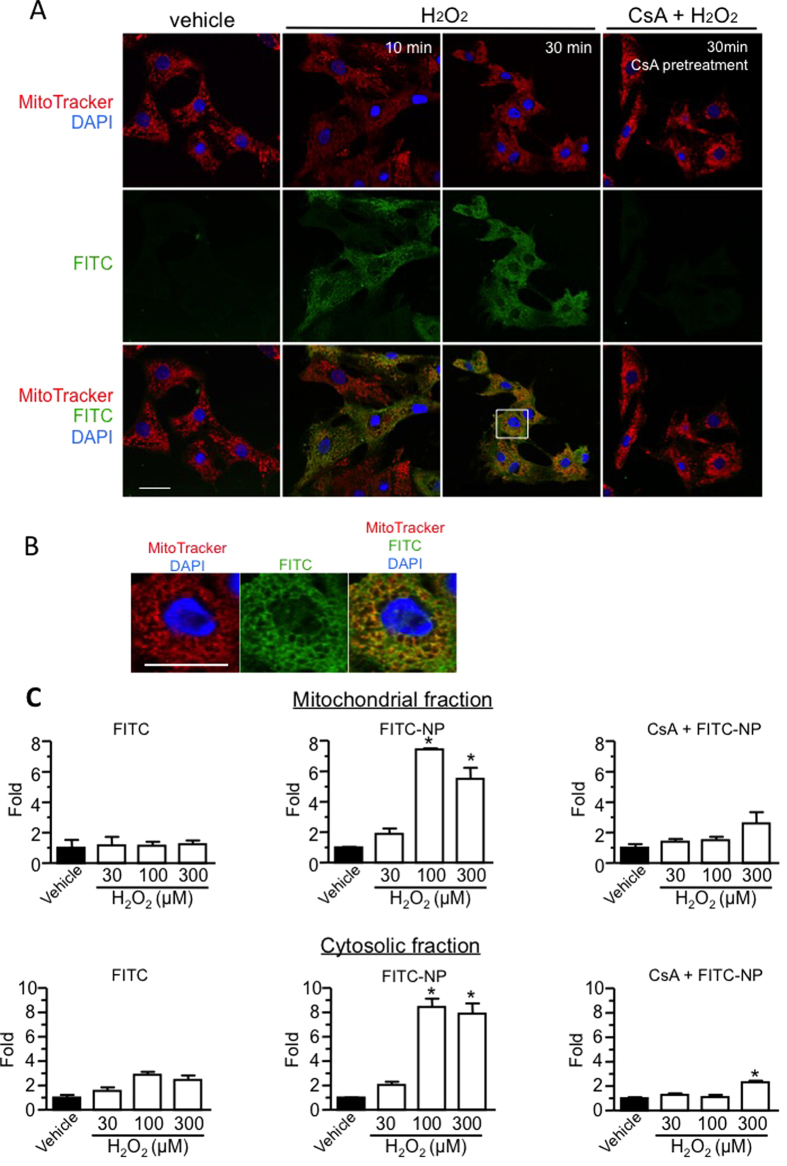
Mitochondria-targeting properties of FITC-NP in neonatal rat ventricular myocytes treated with hydrogen peroxide (H_2_O_2_). (**A**) Subcellular localization of FITC-NP in cardiomyocytes treated with H_2_O_2_. FITC signals were observed in cytosol and mitochondria of cardiomyocytes treated with 100 μM H_2_O_2_. Red represents the mitochondria, green represents the FITC signals, and blue represents the nucleus (DAPI). Pretreatment with cyclosporine A attenuated intracellular uptake of FITC-NP in H_2_O_2_-treated cells. Scale bar: 20 μm. (**B**) Expanded confocal microscopy images of the boxed area in (**A**). (**C**) We measured the FITC concentrations in the mitochondria and cytosol. The data represent the mean ± SEM (N = 3 per bar). *P < 0.05 versus the vehicle control based on a one-way ANOVA followed by Bonferroni’s multiple comparison test.

Neonatal rat ventricular cardiomyocytes rarely internalize FITC-NP spontaneously under normal culture conditions (Fig. 5A). When the cardiomyocytes were treated with FITC-NP and ionomycin (a calcium-selective ionophore that depolarizes the plasma membrane potential), FITC signals were noted in intracellular cytosolic areas, but not in the mitochondria (Supplemental Fig. 4). To represents oxidative stress during IR, the cardiomyocytes were stimulated with hydrogen peroxide (H_2_O_2_); the addition of H_2_O_2_ and FITC-NP increased the intracellular FITC signal in cardiomyocytes, and the FITC signal co-localized with mitochondria, which was inhibited by pretreatment with CsA (Fig. 5A,C).

Because pretreatment with CsA inhibited PLGA nanoparticle delivery to the mitochondria, we investigated the relationship between mPTP opening and the mitochondrial distribution of FITC-NP in H_2_O_2_-treated cardiomyocytes. mPTP opening induced a loss of mitochondrial membrane potential, which preceded irreversible mitochondrial injury.

**Figure 6:**
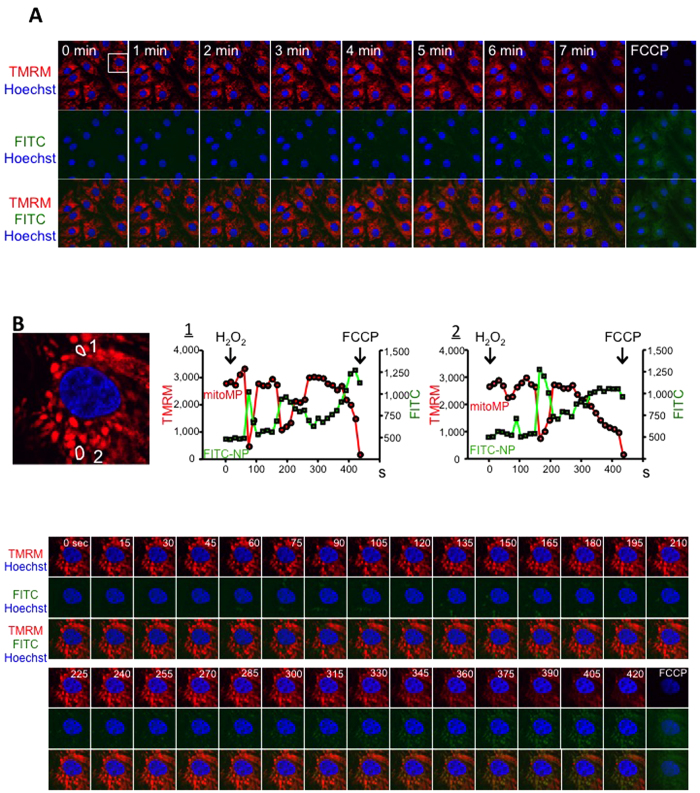
Time-dependent uptake study in neonatal rat ventricular myocytes. (**A**) Confocal images of time-dependent FITC-NP uptake in cardiomyocytes after treatment with 300 μM H_2_O_2_. The loss of TMRM fluorescence in H_2_O_2_-treated cardiomyocytes was exclusively associated with mitochondrial uptake of FITC-NPs. The cells were stained with the mitochondrial membrane potential marker TMRM (red), FITC (green), or Hoechst (blue). (**B**) Expanded confocal microscopy images of a representative cell from the boxed area in (**A**). Time-dependent changes in TMRM fluorescence (red) and FITC fluorescence (green) from two mitochondria in the selected cell are shown here and in a supplemental movie. A reverse relationship was observed between the TMRM and FITC fluorescence findings in the individual mitochondria. The addition of the protonophore carbonylcyanide *p*-trifluoromethoxyphenyl-hydrazone (FCCP) induced full dissipation of the mitochondrial membrane potential.

Tetramethylrhodamine methyl ester (TMRM) is readily sequestered by healthy mitochondria, but its fluorescence is rapidly lost when the mitochondrial membrane potential dissipates. As previously reported^22^,^23^, we detected a transient loss and recovery followed by an irreversible collapse of the mitochondrial membrane potential after H_2_O_2_ treatment. Importantly, under these conditions, we demonstrated a loss of mitochondrial membrane potential was associated with the delivery of FITC-NP to the mitochondrion. However, restoration of the mitochondrial membrane potential separated the FITC-NP from the mitochondrion (Fig. 6 and Supplemental movie), which suggests delivery of PLGA nanoparticles to the mitochondria is associated with mPTP opening.

To determine whether nanoparticle-mediated CsA targeting to the mitochondria exhibits greater cardioprotection than CsA alone, the effects of CsA-NP and CsA on H_2_O_2_-induced cardiomyocyte cell death were examined 3 h after H_2_O_2_ was added to cardiomyocytes (Supplemental Fig. 5). As previously reported^23^, treatment with 200 nM CsA inhibited H_2_O_2_-induced cell death. Importantly, CsA-NP inhibited cell death at lower concentrations (20 nM) than CsA alone.

## Discussion

The novel findings herein are as follows. (1) PLGA nanoparticles were localized to the IR myocardium mitochondria after an intravenous injection at the time of reperfusion in mice; (2) intravenous treatment with CsA-NP at the time of reperfusion enhanced cardioprotection against IR injury by CsA alone through the inhibition of mPTP opening and ameliorated left ventricular remodeling in mice; and (3) PLGA nanoparticle delivery to the mitochondria was associated with mPTP opening under oxidative stress *in vitro*.

Although several nano-size carriers have been generated for drug delivery in cancer cell lines and adipocytes *in vitro*^24^,^25^, no prior studies have shown nanoparticle-mediated drug delivery to the IR myocardium mitochondrion *in vivo*. In the present study, we demonstrated, for the first time, that intravenously injected CsA-NP at the time of reperfusion is a clinically feasible drug delivery system for targeting IR myocardium mitochondria *in vivo*. Nanoparticulation yielded more than an approximately 5-fold greater CsA in the IR myocardium mitochondrial fractions, enhanced the cardioprotective effects of CsA (as indicated by the reduced MI size at a lower CsA concentration) and ameliorated left ventricular dysfunction, as well as remodeling in a mouse IR model. From *in vitro* drug release study, approximately 50% of cyclosporine was released to physiological solution within 6 hours (Supplementary Fig. 2). In translational medicine, PLGA nanoparticles have been used as drug carriers with biosafety approval for human use by the US Food and Drug Administration^19^,^26^, the European Medicine Agency (EMA) and the Japanese regulatory agency (PMDA). We have initiated a phase I/IIa clinical trial at Kyushu University Hospital (UMIN000008011) to investigate the safety and efficacy of intramuscular injections of PLGA nanoparticles incorporated with pitavastatin in ischemic limbs of patients with critical limb ischemia. We have also completed a phase I clinical trial at Kyushu University Hospital (UMIN 000014940) to investigate the safety of a single intravenous infusion of PLGA nanoparticles incorporated with pitavastatin in healthy volunteers. CsA has been clinically approved and widely used for the treatment of immune diseases. Therefore, CsA-NP can be developed as a new therapeutic agent for myocardial IR injury with high efficacy and safety that is readily translated into clinical trials.

Herein, we demonstrated that cardioprotection by CsA-NP was associated with the inhibition of mPTP opening-dependent cytochrome c leakage from mitochondria to the cytosol 30 min after IR and was attenuated in cyclophilin D^−/−^ mice, which indicates the prevention of mPTP opening inhibition is a primary mechanism that underlies cardioprotection by CsA-NP. As we and other groups have previously reported^14^,^18^, nanoparticles are also taken up by mononuclear phagocytic systems, including circulating neutrophils and monocytes, and are recruited to IR myocardia. In the present study, CsA-NP did not reduce leukocyte or Ly6C^high^ inflammatory monocyte recruitment to IR myocardia or the chemotaxis activity of bone marrow-derived macrophages. In addition, *in vivo* FMT and *ex vivo* FRI imaging analyses revealed that CsA-NP did not inhibit inflammatory protease activity but did limit IR injury. These data suggest the observed therapeutic effects induced by CsA-NP are not mediated by inflammation-related cardiomyocyte death.

We^9^,^10^,^12^,^13^,^27^ and other groups^28^ have reported that many types of cells, including endothelial and smooth muscle cells, spontaneously internalize PLGA nanoparticles under normal culture conditions via endocytosis. However, in the present *in vitro* experiments, primary cultured rat cardiomyocytes rarely internalize the nanoparticle spontaneously, which suggests a substantial contribution from electrostatic repulsion between the anionic nanoparticles and cardiomyocyte anionic plasma membranes. This repulsion may interrupt spontaneous internalization of anionic PLGA nanoparticles via ordinary endocytotic processes. Interestingly, we observed increased intracellular PLGA nanoparticle delivery to the cardiomyocyte cytosol and mitochondria following the addition of H_2_O_2_ *in vitro*, which was inhibited by the pretreatment with CsA *in vitro*. Confocal microscopic analyses (Figs 5, 6 and Supplemental movie) revealed that H_2_O_2_-induced dynamic loss/recovery of the mitochondrial membrane potential was closely associated with PLGA nanoparticle delivery/detachment to the mitochondria. It has been reported in cardiomyocytes that (1) oxidative stress in the early phase of IR mediates mPTP opening^1^,^29^, and (2) transient mPTP opening initiates extensive, unconventional endocytic responses during IR *in vivo* and hypoxia/reoxygenation *in vitro*^30^,^31^. In addition, pharmacokinetic studies suggest PLGA nanoparticles modulate the *in vivo* kinetics of FITC or CsA through selective delivery to the IR cardiomyocyte cytosol and mitochondria in the area at risk. Electron microscopic analyses identified PLGA nanoparticles in the vicinity of the IR cardiomyocyte mitochondria. Overall, these data suggest the importance of the dynamic mPTP opening and the resulting loss of mitochondrial membrane potential that underlies the mitochondrial delivery mechanism for drugs to the IR myocardium using PLGA nanoparticles.

In conclusion, PLGA nanoparticle-mediated CsA targeting to the IR myocardium mitochondria enhanced cardioprotection against IR injury. Targeting mPTP opening with the use of CsA-NP may offer a new, more effective therapeutic mechanism for protecting organs from IR injury in patients with AMI and other clinical settings, such as cardiac surgery or recovery from cardiac arrest after an IR injury in multiple organs.

## Methods

Additional details of the experimental procedures are included in the online-only Data Supplement.

### PLGA Nanoparticle Preparation

PLGA, which has an average molecular weight of 20,000 and a copolymer ratio 75:25 lactide to glycolide (Wako Pure Chemical Industries Ltd., Osaka, Japan), was used as a matrix for the nanoparticles; polyvinyl alcohol (PVA-403; Kuraray, Osaka, Japan) was used as a dispersing agent. We prepared PLGA nanoparticles that incorporated the fluorescent marker fluorescein-isothiocyanate (FITC; Dojin Chemical, Tokyo, Japan) (FITC-NP) or CsA (Sigma Aldrich, MO) (CsA-NP) using an emulsion solvent diffusion method as previously described^9^,^12^,^15^. The FITC-NP contained 4.06% (wt/vol) and FITC, and the CsA-NP contained 2.67% (wt/vol) CsA. The surface charges (zeta potential) were also analyzed using a Zetasizer Nano (Sysmex, Hyogo, Japan) and were −20.3 mV (FITC-NP) and −20.2 mV (CsA-NP). The physical properties (monodispersity) of CsA-NP and FITC-NP are shown in Supplemental Fig. 6 and Supplemental Table 6.

### Mouse myocardial IR model

The study protocol was reviewed and approved by the committee on the Ethics of Animal Experiments, Kyushu University Faculty of Medicine and was conducted in accordance with the American Physiological Society guidelines. Male C57BL/6J and cyclophilin D^−/−^ mice were purchased from Jackson Laboratories (Stock#: 009071, Bar Harbor, ME). The animals were maintained on a 12-h light-dark cycle with free access to normal chow and water. The murine model for myocardial IR injury was based on previously described methods^32^. Adult male mice (Age: 10–12 weeks, BW: 20–30 g) were anesthetized via an intraperitoneal injection of pentobarbital sodium (60 mg/kg) and maintained using 1% isoflurane with a ventilator after intubation. The heart was exposed via a left thoracotomy on a heated board. Transient myocardial ischemia was produced through ligation of the anterior descending branch of the left coronary artery (LAD) using an 8–0 silk suture with a silicon tube placed alongside the LAD. Regional ischemia was confirmed through ECG changes (ST elevation). After reperfusion, the chest was closed, and animals were allowed to recover from the surgery.

### Experimental protocols

#### Experimental protocol 1

At the time of reperfusion, the animals were divided into three groups, which received intravenous injections of the following drugs: 1) vehicle (5.0 mL/kg saline), 2) FITC alone (FITC 0.06 mg in 5.0 mL/kg saline), or 3) FITC-NP (PLGA 1.48 mg that contained 0.06 mg FITC in 5.0 mL/kg saline). Certain animals were euthanized 5 min after reperfusion to examine the FITC signals in the subcellular mitochondrial and cytosolic fractions and 3 h after reperfusion to examine the FITC fluorescence in the myocardium.

#### Experimental protocol 2

At the onset of reperfusion, the mice were divided into four groups; each group received an intravenous injection of the following drugs: 1) vehicle (5.0 mL/kg saline), 2) FITC-NP (1.4 mg PLGA in 5.0 mL/kg saline), 3) CsA alone (1.0, 2.5, 10, or 25 mg/kg in 5.0 mL/kg saline), or 4) CsA-NP (PLGA that contained 1.0, 2.5, 10, or 25 mg/kg cyclosporine in 5.0 mL/kg saline). The animals were euthanized, the myocardial tissue samples were collected for immunoblotting 30 min after reperfusion, and the infarct size was measured 24 h after reperfusion. The CsA concentrations in the whole blood and tissue homogenates from various organs were measured 5 min, 30 min, 3 h, and 24 h after the intravenous administration of 2.5 mg/kg CsA or CsA-NP that contained 2.5 mg/kg CsA. Additionally, the CsA concentrations of mitochondrial fractions from the myocardium were examined 5 min and 30 min after the intravenous administration of 2.5 mg/kg CsA or CsA-NP that contained 2.5 mg/kg CsA.

#### Experimental protocol 3

Cyclophilin D^−/−^ mice and control wild-type mice were subjected to 45 min of ischemia followed by reperfusion. The mice were divided into the following three groups, which received an intravenous injection of the following drugs: 1) vehicle (5.0 mL/kg saline) at the time of reperfusion, 2) CsA-NP (PLGA that contained 1.0 mg/kg cyclosporine in 5.0 mL/kg saline) at the onset of reperfusion, or 3) erythropoietin at 2500 U/kg 24 h and 30 min prior to the induction of myocardial ischemia^20^. Twenty-four h after reperfusion, the animals were euthanized, and the MI was measured.

### Mouse Heart Mitochondria Isolation

The mouse heart mitochondria were isolated in accordance with the manufacturer’s protocol (Abcam, MA). The purity of the subcellular preparation was assessed through immunoblotting^33^.

### Cyclosporine concentration measurements

The mice were subjected to 30 min of ischemia followed by reperfusion and were intravenously injected with 2.5 mg CsA or CsA-NP that contained 2.5 mg/kg CsA at the onset of reperfusion. Blood samples were collected in tubes with EDTA-2K (Sigma-Aldrich), and the organs (brain, heart, liver, kidney, spleen, and lung) were collected from individual mice at predetermined time points. The mitochondria were isolated from ischemic and non-ischemic myocardia in separate experiments. All samples were weighed and homogenized. The CsA concentrations in whole-blood and tissue homogenates of collected samples were measured by a previously established and validated radioimmunoassay^34^, which correlates highly with a validated HPLC method.

### Western Blot Analysis

Homogenates of ischemic and non-ischemic myocardium were analyzed by immunoblotting. At 30 min after IR, protein was extracted from the subcellular mitochondrial or cytosolic fractions from the ischemic and non-ischemic heart samples. The samples were solubilized in lysis buffer, and proteins (mitochondria: 2.0 μg, cytosol: 5.0 μg) were separated on 4–20% SDS-polyacrylamide gels and then blotted to PVDF membranes.

### Cardiomyocyte preparation and culture

A primary neonatal rat ventricular myocyte culture was prepared using neonatal Sprague-Dawley rat ventricles as previously described^35^.

### Cell treatment

The cells were washed with HBSS and loaded with 250 nM MitoTracker Orange CMTMRos (Invitrogen, CA) in a culture medium for 30 min. In certain groups, the cells were pretreated with 2 μM CsA for 30 min. The culture medium was changed and treated with a vehicle or 100 μM H_2_O_2_ for 10 or 30 min. The cells were washed with phosphate-buffered saline, and fresh medium with FITC-NP that contained 10 μM FITC was added for 30 min; the cells were washed again, fixed with methanol at −20 °C for 20 min, mounted with the medium that contained DAPI (Vectashield, Vector Laboratories, CA).

### Time-lapse imaging

Neonatal rat ventricular myocytes were loaded with 200 nM TMRM (ImmunoChemistry Technologies, MN) and 10 μM Hoechst 33342 (Dojin Chemical) for 15 min. To minimize cell contraction, time-lapse imaging was performed in a Ca^2+^-free medium. The experiments were performed using a stage-top incubator (Tokai Hit, Shizuoka, Japan) at 37 °C in humidified air with 5% CO_2_. FITC-NP that contained 10 μM FITC and 300 μM H_2_O_2_ were added, and images were collected every 15 s.

### Cell viability

The CTB assays (Promega, WI) were performed in accordance with the supplier’s protocol.

### Chemotaxis assay

We obtained bone marrow-derived macrophages (BMDMs) and measured the chemotactic activity of BMDMs as previously described^36^.

### Statistical Analysis

The data are expressed as the mean ± SEM. We analyzed the differences between the two groups using unpaired *t*-tests; the differences among 3 groups or more were assessed using an ANOVA and post-hoc Bonferroni’s multiple comparison tests with Prism Software version 4.0 (Graph Pad Software, CA). P values < 0.05 were considered statistically significant.

## Additional Information

**How to cite this article**: Ikeda, G. *et al*. Nanoparticle-Mediated Targeting of Cyclosporine A Enhances Cardioprotection Against Ischemia-Reperfusion InjuryThrough Inhibition of Mitochondrial Permeability Transition Pore Opening. *Sci. Rep.* **6**, 20467; doi: 10.1038/srep20467 (2016).

## Supplementary Material

Supplementary Information

Supplementary Movie
