# Supplementary material for: Nanoparticle-Mediated Targeting of Cyclosporine A Enhances Cardioprotection Against Ischemia-Reperfusion Injury Through Inhibition of Mitochondrial Permeability Transition Pore Opening
Source: Sci Rep. 2016 Feb 10;6:20467. doi: 10.1038/srep20467 (PMC4748220; doi:10.1038/srep20467)
Supplement: Supplementary Information [file srep20467-s1.doc]

**SUPPLEMENTAL MATERIAL**

**Nanoparticle-Mediated Targeting of Cyclosporine A**

**Enhances Cardioprotection Against Ischemia-Reperfusion Injury**

**Through Inhibition of Mitochondrial Permeability Transition Pore Opening**

Gentaro Ikeda, M.D.; Tetsuya Matoba, M.D., Ph.D.; Yasuhiro Nakano, M.D.; Kazuhiro Nagaoka, M.D.; Ayako Ishikita, M.D.; Kaku Nakano, Ph.D.; Daiki Funamoto, Ph.D.; Kenji Sunagawa, M.D., Ph.D.; Kensuke Egashira, M.D., Ph.D. ^*^

**Supplemental Methods**

**Determination of MI size**

At 24 h after reperfusion, the animals were reanesthetized and intubated, and the chest was opened. The LAD was re-ligated with the same suture that had been left at the site of the ligation. To demarcate the ischemic area at risk (AAR), 2% Evans blue dye (Sigma Aldrich, MO) was injected via the inferior vena cava. The heart was then excised and sliced into sequential five 1.0-mm-thick cross sections. The sections were incubated with 2% 2,3,5-triphenyltetrazolium chloride (TTC, Sigma Aldrich) in saline for 10 min at 37°C and were photographed with a stereomicroscope (HC-2500, Nikon). The MI area (TTC-negative, white), non-MI area within the AAR (TTC-positive/Evans blue-negative, red), non-ischemic area (TTC-positive/Evans blue-positive, purple), and AAR (Evans blue-negative) were analyzed using ImageJ software (version 1.44, National Institutes of Health, http://rsb.info.nih.gov/ij/).

**Mouse Heart Mitochondria Isolation**

The heart was quickly excised, minced on ice, resuspended in 1 mL of washing buffer, and homogenized using a glass Dounce homogenizer. The homogenate was centrifuged at 1000×*g* and 4°C for 5 min. The supernatant was re-centrifuged at 12,000×*g* for 15 min to pellet the mitochondria, and the resultant supernatant was used as the supernatant fraction. The pellet was washed twice with isolation buffer. The purity of the subcellular preparation was assessed through immunoblotting. Briefly, equal quantities of the protein from each subcellular fraction were probed through immunoblotting with antibodies for GAPDH or VDAC. Highly pure mitochondria did not exhibit a GAPDH signal, and highly pure cytosol did not exhibit a VDAC signal after several min of exposure.

**Antibodies for western blot analysis**

The following antibodies were used as primary antibodies: cytochrome c (supernatant fraction, 1/1000; mitochondrial fraction, 1:2000; Santa Cruz Biotechnology, CA), Bax (1:1000, Cell Signaling Technology, MA), GAPDH (1:2500, Santa Cruz Biotechnology), and VDAC (1:1000, Cell Signaling Technology).

**Transmission Electron Microscopy**

The animals were intravenously injected with FITC-NP at the time of reperfusion and perfused with a fixative that contained fresh 2.5% glutaraldehyde in 0.1 M phosphate buffer (pH 7.4, 4°C) 30 min after reperfusion. The myocardial samples were obtained from the ischemic (anterior lesion) and non-ischemic (septal lesion) myocardium, and the sliced samples were fixed overnight. These small blocks of heart samples were then post-fixed in 2% OsO4 in 0.1 M phosphate buffer (pH 7.3, 4.0°C) for 2 h. The specimens were dehydrated using a graded ethanol series. Ultrathin sections were then cut at 80 nm using a diamond knife on an ultramicrotome (Reichert, NY, USA). The sections were collected on 150 mesh copper grids and stained with uranyl acetate and lead citrate. The specimens were then observed using an HT7700 transmission electron microscope (Hitachi, Tokyo, Japan). The accelerating voltage was 80 kV.

**Echocardiography**

At predetermined time points, a transthoracic two-dimensional echocardiography was performed using a 12-mHz probe (Visualsonics, Ontario, Canada) on mice anesthetized via the inhalation of isoflurane (1-1.5%) and maintained with a heart rate at approximately 500 bpm. M-mode interrogation was performed using the parasternal short-axis perspective at the greatest LV end-diastolic dimension (LVEDD). The LVEDD and left ventricular end-systolic dimension (LVESD) were determined and used to calculate the percent of fractional shortening (FS) and ejection fraction (EF). The FS was calculated using the following formula: [(LVEDD-LVESD) / LVEDD]×100. The EF was calculated using the following formula: [(LVEDV- LVESV) / LVEDV]×100, where the EDV is the end-diastolic volume, and the ESV is the end-systolic volume.

**Blood Pressure Measurements**

The blood pressure was monitored at predetermined time points using a noninvasive tail cuff method.

**Flow cytometry**

The peripheral blood was drawn via cardiac puncture, and the erythrocytes were lysed using VersaLyse Lysing solution for 10 min at room temperature. The hearts were removed and digested using a cocktail composed of 450 U/mL collagenase type I, 125 U/mL collagenase type XI, 60 U/mL DNase I and 60 U/mL hyaluronidase (all enzymes were obtained from Sigma-Aldrich) in PBS that contained 20 mM HEPES at 37°C for 1 h. The cell suspension was centrifuged at 300 ×*g* and 4°C for 5 min. After blocking the Fc receptor with anti-CD16/32 mAb (BD Biosciences, CA) for 5 min at 4°C, the cell suspensions were incubated with a cocktail composed of mAb against T cells (CD90-PE, 53-2.1), B cells (B220-PE, RA3-6B2), NK cells (CD49b-PE, DX5 and NK1.1-PE, PK136), granulocytes (Ly6G-PE, 1A8), myeloid cells (CD11b-APC, M1/70), and monocyte subsets (Ly6C-FITC, AL-21) (BD Biosciences) for 1 h at 4°C. All leukocytes were then analyzed using FACS Gallios (BD Biosciences). The leukocytes were also incubated with the appropriate isotype control (BD Biosciences). The monocytes/macrophages were identified as CD11b^high^ (CD90/B220/CD49b/NK1.1/Ly6G)^low^ Ly6C^high/low^, the neutrophils were identified as CD11b^high^ (CD90/B220/CD49b/NK1.1/Ly6G)^high^, and the lymphocytes were identified as CD11b^low^ (CD90/B220/CD49b/NK1.1/Ly6G)^high^ as previously described.[^1^](#_ENREF_1)

**Fluorescence molecular tomography (FMT) and fluorescence reflectance imaging (FRI)**

Five nanomoles of pan-cathepsin protease sensor (ProSence 680, PerkinElmer, MA) and 2 nM of cellular death sensor (Annexin-Vivo 750, PerkinElmer) were intravenously administered at the onset of reperfusion and 22 h after reperfusion, respectively. Twenty-four h after reperfusion, the animals were scanned using an FMT-2000 system (PerkinElmer). The volume of interest (ROI) for the heart was positioned based on a previous report.[^2^](#_ENREF_2) After the animals were imaged *in vivo*, they were sacrificed, and the heart was removed. The heart was cut into sequential 1-mm-thick cross sections, stained with triphenyltetrazolium chloride (TTC) and imaged using fluorescence reflectance imaging with FMT-2000’s planar imaging capability.

**Cardiomyocyte preparation and culture**

The neonatal rats were euthanized under anesthesia with isoflurane, and the hearts were rapidly excised and digested. After the myocardial tissue was digested with trypsin (Wako Pure Chemical, Osaka, Japan) and collagenase type 2 (Worthington, NJ), the cells were suspended in Dulbecco's Modified Eagle's Medium (Sigma-Aldrich) with 10% fetal bovine serum (FBS, Thermo Scientific, MA), penicillin (Invitrogen, CA), and streptomycin (Invitrogen). The cells were plated twice in 100-mm culture dishes (Cellstar, Greiner Bio-One, NC) for 70 min each to reduce the non-myocytes. Non-adherent cells were plated in culture dishes (Primaria, Falcon) (BD Biosciences, CA) or glass-bottom dishes (Matsunami, Japan) at an appropriate density for each experiment. The myocytes were maintained at 37°C in humidified air with 5% CO_2_ for 36 h after they were plated on culture dishes.

**Quantification of nanoparticles in the intracellular compartments**

Neonatal rat ventricular myocytes (5 x 10^6^ cells/ml) were treated with a vehicle; 30, 100, or 300 µM H_2_O_2_ for 30 min followed by treatment with FITC (10 µM) or FITC-NP (that contained 10 µM of FITC) for 30 min. The mitochondria and cytosol fractions were isolated using a mitochondria isolation kit for mammalian cells (Abcam, MA); we quantified the FITC per nanogram for each protein isolated.

**Confocal Imaging**

A Nikon A1 confocal microscope (Nikon, Tokyo, Japan) was used for neonatal rat ventricular myocyte imaging. Hoechst and DAPI were excited at 405 nm, MitoTracker Orange CMTMRos and tetramethylrhodamine methyl ester (TMRM) were excited at 561 nm, and FITC was excited at 457 nm. The fluorescence light emitted was collected by 2 photomultiplier tubes fitted with band-pass filters for 425 to 475 nm (for Hoechst and DAPI), 565 to 615 nm (for TMRM and MitoTracker Orange CMTMRos), and 500 to 530 nm (for FITC). The images were generated using a 60x oil immersion objective. For multi-color imaging, each excitation wavelength was applied, and we individually collected the emission by switching between fluorescence channels.

**Calcineurin activity assay**

The calcineurin activity was determined using a Biomol Green™ Quantizyme™ Assay System (Calcineurin Cellular Assay Kit Plus AK-816, Biomol International, LP) in accordance with the manufacturer’s protocol. Briefly, the frozen tissue was homogenized in ice-cold lysis buffer (50 mM Tris, pH 7.5, 0.1 mM EDTA, 0.1 mM EGTA, 1 mM DTT, and 0.2% NP-40) that contained a protease inhibitor. The samples were centrifuged at 100,000×*g* and 4°C for 45 min, and the supernatant was collected. The free phosphate in the supernatant was removed by passing the supernatant through a desalting column (P6 DG desalting resin) equilibrated with lysis buffer. The extracts collected (5 µg in each sample) were used to determine the calcineurin activity using a calcineurin-specific RII phosphopeptide.

**Cell viability**

The cell viability was measured using Cell Titer Blue (CTB) assays (Promega, WI). Briefly, cardiac myocytes (1x10^5^/100 µl) were seeded onto 96-well dishes. We added CsA or CsA-NP 10 min prior to the H_2_O_2_ treatment. The cell viability was measured 3 h after a treatment with 300 µM H_2_O_2_.

**Chemotaxis assay**

Bone marrow-derived macrophages (BMDMs) were obtained through culturing bone marrow cells from 8-week-old C57BL/6 mice in RPMI 1640 supplemented with 20% FBS, 40 ng/ml M-CSF (R&D Systems, MN) and 2 mM UltraGlutamine (Lonza Japan, Chiba, Japan) at 37°C in a 5% CO_2_ environment. Six days later, the BMDMs were collected and plated; the medium was replaced with a starvation medium that contained 0.02 to 2 µM of CsA, nanoparticles that contained 0.02 to 2 µM of CsA, FITC-NP or a vehicle alone for 90 min. We measured the chemotactic activity of BMDM in response to 10 or 100 ng/mL MCP-1 in a 96-well microchemotaxis Boyden chamber (ChemoTx; Neuroprobe) as previously described.[^3^](#_ENREF_3) The macrophages that transmigrated through the micropore were stained with trypan blue, and we counted the number of BMDMs that migrated in response to MCP-1.

**In vitro release kinetics of cyclosporine from nanoparticles**

To measure release kinetics of cyclosporine, CsA-NP (n = 3) was dissolved in PBS, and the released cyclosporine from nanoparticles was measured by radioimmunoassay at predetermined time points.

**Supplemental Figures and Figure Legends**


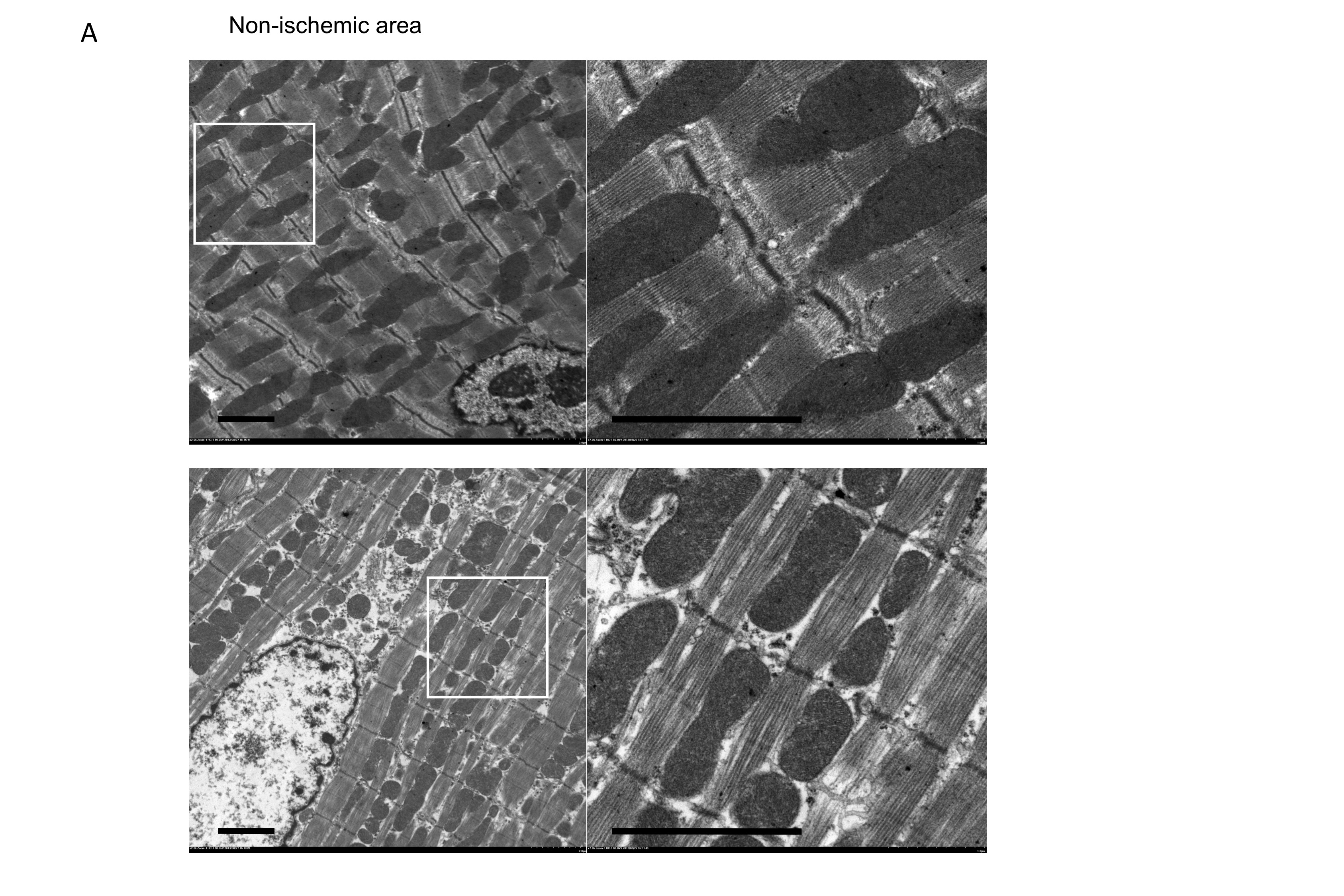


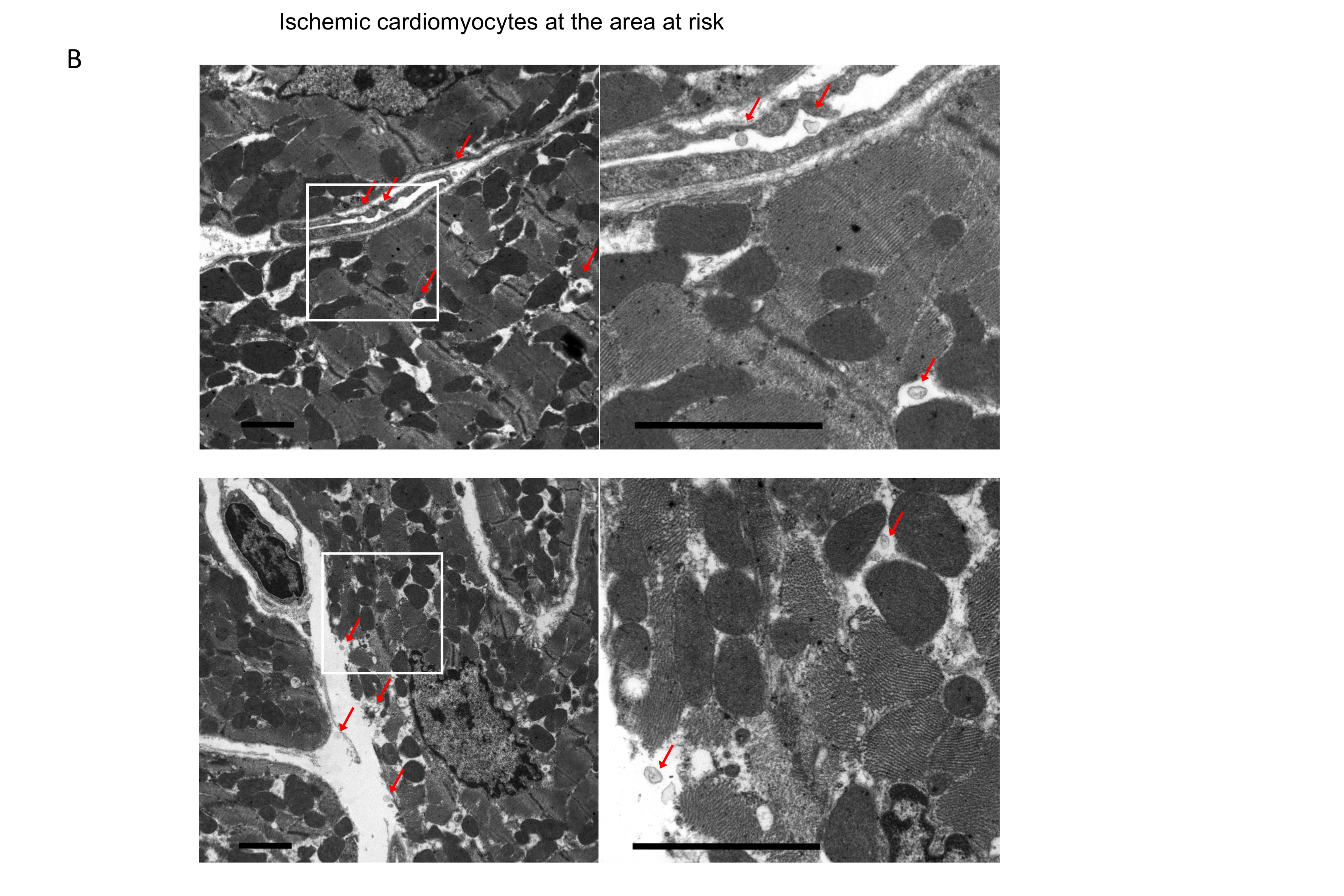


**
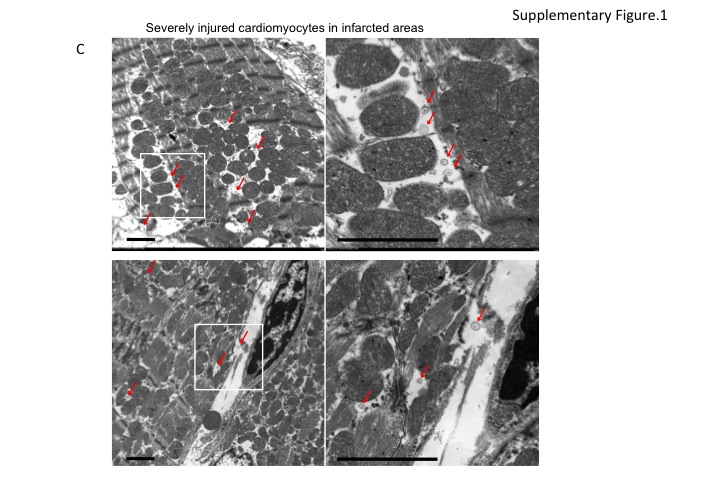
Supplemental Figure 1.** Electron microscopy study.

Electron microscopy images from the non-ischemic myocardium (**A**), ischemic cardiomyocytes in the area at risk (**B**), and severely injured cardiomyocytes in the infarcted areas (**C**).

Red arrows = nanoparticle. Scale bar = 2 µm.

**
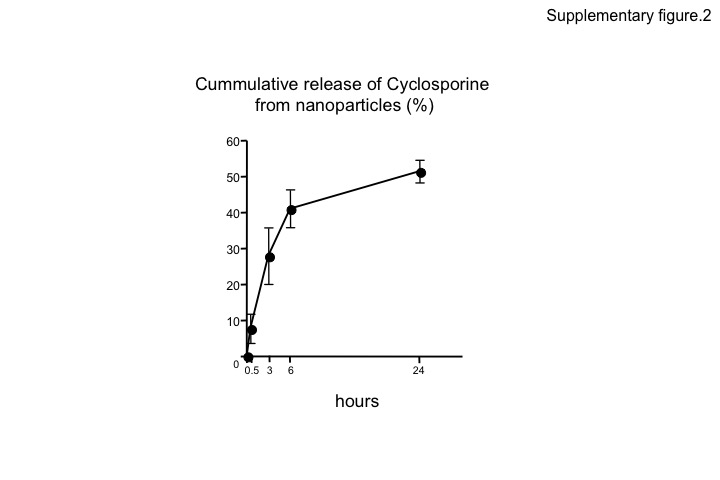
**

**Supplementary Figure 2.** *In vitro* time course of cumulative cyclosporine release from the CsA-NP (*n* = 3). The percentage of incremental quantities of released Cyclosporine were plotted against time.

**
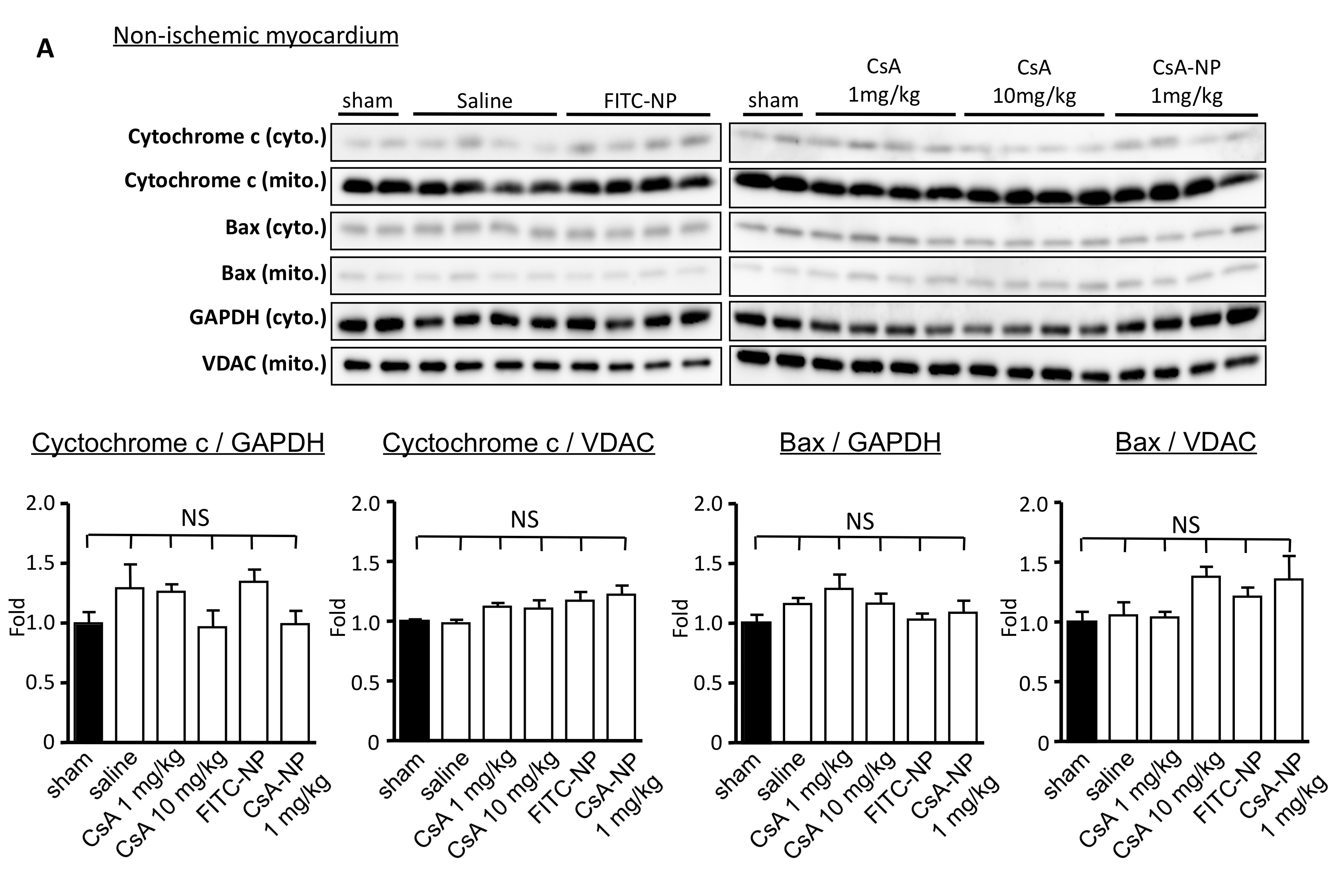
**

**Supplemental Figure 3.** CsA-NP effects on cytochrome c leakage in non-ischemic myocardia. **A.** Mitochondrial and cytosolic fractions in non-ischemic myocardia were analyzed through immunoblotting for mitochondrial Bax recruitment and cytochrome c release into the cytosol in the sham, saline, FITC-NP, 1.0 mg/kg CsA, 10 m/kg CsA, and CsA-NP that contained 1.0 mg/kg CsA groups. **B to E.** The bar graphs show the normalized fold changes in cytochrome c leakage into the cytosol and Bax translocation to the mitochondria. The data are expressed as the mean ± SEM (N=8 each) and were compared using a one-way ANOVA followed by Bonferroni’s multiple comparison test.

**
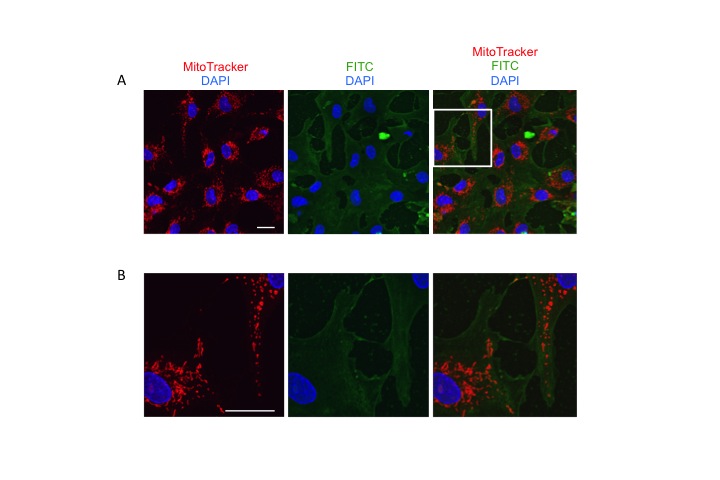
**

**Supplemental Figure 4.** Subcellular localization of FITC-NP in cardiomyocytes treated with ionomycin.

**A.** FITC signals were noted in intracellular cytosolic areas, but not in the mitochondria. Scale bar: 20 µm.

**B.** Expanded confocal microscopy images of the boxed area. Scale bar: 20 µm.

**
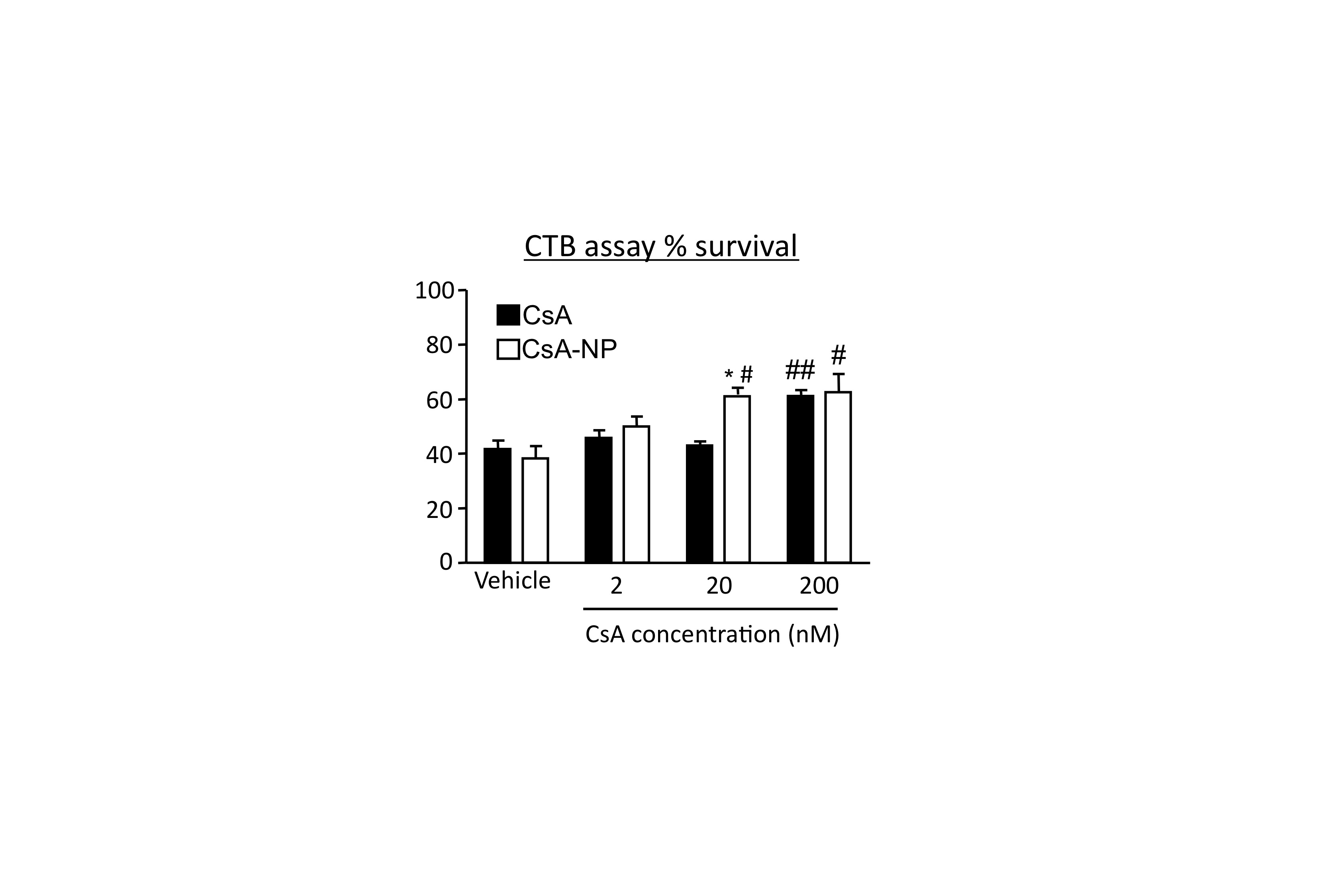
**

**Supplemental Figure 5.** A Cell Titer Blue assay was used to determine cardiomyocyte cell death induced by 300 µM hydrogen peroxide (H_2_O_2_). The data represent the mean ± SEM (N=3 per bar). *P<0.05 versus cyclosporine A (CsA); and #P<0.05, ##P<0.01 versus vehicle according to a two-way ANOVA followed by Bonferroni’s multiple comparison test.

**
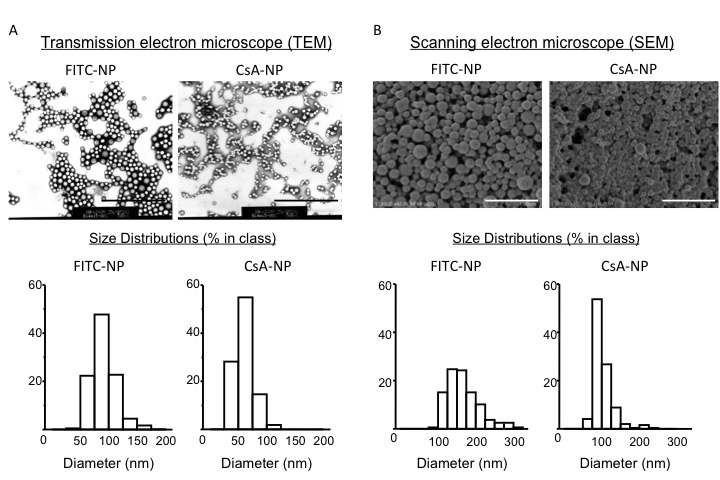
**

**Supplemental Figure 6.** The physical properties of FITC-NP and CsA-NP.

1. TEM pictures and particle size distribution. Scale bar: 500 µm. B. SEM pictures and particle size distribution. Scale bar: 1 µm.
2. **Supplemental Movie.** In H_2_O_2_-treated cardiomyocytes, mitochondrial targeting (FITC positivity) was exclusively observed in the mitochondria with negative TMRM fluorescence.

**Supplemental Table 1**. The positive and negative cardioprotective effects of cyclosporine A on myocardial ischemia-reperfusion injury (myocardial infarct size) in animals and humans.

| **Author** | **Model** | **Dose (mg/kg)** | **Time (min) before reperfusion** | **Outcome** |
| --- | --- | --- | --- | --- |
| Gomez et al.[^4^](#_ENREF_4)  Kerstin et al.[^5^](#_ENREF_5)  Lim et al.[^6^](#_ENREF_6)  Dow and Kloner[^7^](#_ENREF_7)  Huhn et al.[^8^](#_ENREF_8)  Liu et al.[^9^](#_ENREF_9)  Argaud et al.[^10^](#_ENREF_10)  Pagel and Krolikowski[^11^](#_ENREF_11)  Krolikowski et al.[^12^](#_ENREF_12)  Karlsson et al.[^13^](#_ENREF_13)  Karlsson et al.[^14^](#_ENREF_14)  Lie et al.[^15^](#_ENREF_15)  Skyschally et al.[^16^](#_ENREF_16)  Piot et al.[^17^](#_ENREF_17)  Ghaffari et al.[^18^](#_ENREF_18)  Cung et al.[^19^](#_ENREF_19) | Mouse  Mouse  Mouse  Rat  Rat  Rat (3-5 months)  Rat  (20-24 months)  Rabbit  Rabbit  Rabbit  Pig  Pig  Pig  Pig  Human  Human  Human | 10  10  10  5  10  5  10  10  10  5  5  10  2.5  10  10  5  2.5  2.5  2.5 | 5  5  0  2  2  5  5  5  1  5  5  5  7  N/A  5  5  < 10  1  < 10 | Positive  Positive  Positive  Negative  Negative  Positive  Positive  Negative  Positive  Negative  Negative  Positive  Negative  Negative  Negative  Positive  Positive  Negative  Negative |

N/A: not available

**Supplemental Table 2**. Cyclosporine concentrations in various organs following the intravenous administration of cyclosporine A (CsA) at 2.5 mg/kg or nanoparticles incorporated with 2.5 mg/kg CsA (CsA-NP).

| Organs/Groups | Time after intravenous administration | | | |
| --- | --- | --- | --- | --- |
|  | 5 min | 30 min | 3 h | 24 h |
| Whole blood (ng/mL)  CsA  CsA-NP | 5,370 ± 1,015  4,188 ± 617 | 1,518 ± 168  1,730 ± 100 | 494 ± 23  952* ± 167 | 39 ± 9  124 ± 52 |
| Brain (ng/g・tissue)  CsA  CsA-NP | 134 ± 22  75 ± 19 | 79 ± 16  61 ± 12 | 98 ± 25  65 ± 14 | 26 ± 11  22 ± 9 |
| Lung (ng/g・tissue)  CsA  CsA-NP | 8,064 ± 1,584  10,325 ± 2,060 | 3,995 ± 649  8,011 ± 1,648* | 1,125 ± 152  2,031 ± 304* | 160 ± 73  123 ± 59 |
| Liver (ng/g・tissue)  CsA  CsA-NP | 13,085 ± 1,082  9,755 ± 1,101 | 8,100 ± 569  10,090 ± 1,247 | 3,547 ± 241  5,169 ± 512* | 529 ± 141  596 ± 69 |
| Spleen (ng/g・tissue)  CsA  CsA-NP | 6,879 ± 1,104  9,830± 1,564 | 6,721 ± 1,057  11,173 ± 752* | 2,345 ± 175  5,438 ± 1,245* | 1,225 ± 434  275 ± 25 |
| Kidney (ng/g・tissue)  CsA  CsA-NP | 12,171 ± 1,208  10,117 ± 1,004 | 8,862 ± 907  8,447± 1,014 | 2,036 ± 231  4,885 ± 279* | 252 ± 35  195 ± 41 |

The data are expressed as the mean ± SEM (N=6 each). **P*<0.05 versus CsA group by unpaired *t*-test.

Values below the measurable limits were replaced with the one-half value of the limits. The analyses of the differences between two groups, in which one value was replaced by the complementary value, were assessed using unpaired *t*-tests following the transformation of the values into a natural logarithm.

|  | **Saline (N=4)** | **CsA at 1.0 mg/kg (N=6)** | **CsA at 10 mg/kg (N=6)** | **CsA-NPs at 1.0 mg/kg (N=6)** |
| --- | --- | --- | --- | --- |
| Calcineurin activity  (nmol PO_4_) | 0.04 ± 0.05 | 0.06 ± 0.05 | 0.16 ± 0.04 | 0.06 ± 0.03 |

**Supplemental Table 3**. Calcineurin activity in the myocardium 3 h after reperfusion.

The data represent the mean ± SEM. The data were compared using a one-way ANOVA followed by Bonferroni’s multiple comparison test.

**Supplemental Table 4**. The effects of cyclosporine A (CsA) or nanoparticles incorporated with CsA (CsA-NP) on cardiac remodeling measured using echocardiography.

|  | | **Weeks after IR** | | | |
| --- | --- | --- | --- | --- | --- |
| **Groups** | | **Baseline** | **1 week** | **2 weeks** | **4 weeks** |
| Saline | LVEDD, mm  LVEDS, mm  EF, %  FS, % | 3.6 ± 0.1  2.4 ± 0.1  62 ± 2  33 ± 1 | 4.3 ± 0.1*  3.5 ± 0.1*  40 ±2*  20 ± 1* | 4.6 ± 0.1*  3.8 ± 0.1*  36 ± 4*  17 ± 2* | 4.6 ± 0.1*  3.9 ± 0.1*  33 ± 2*  16 ± 1* |
| CsA at 1.0 mg/kg | LVEDD, mm  LVEDS, mm  EF, %  FS, % | 3.6 ± 0.1  2.5 ± 0.1  61 ± 1.5  32 ± 1 | 4.3 ± 0.1*  3.5 ± 0.1*  39 ± 3.0*  19 ± 2* | 4.6 ± 0.1*  3.7 ± 0.1*  40 ± 2.4*  19 ± 1* | 4.5 ± 0.1*  3.8 ± 0.1*  32 ± 2.6*  15 ± 1* |
| CsA at 10 mg/kg | LVEDD, mm  LVEDS, mm  EF, %  FS, % | 3.7 ±0.1  2.52 ± 0.1  59.8 ± 1  31.2 ± 1 | 4.1 ± 0.1*†  3.08 ± 0.1*†  49.3 ± 1*†  24.6 ± 1* | 4.2 ± 0.1*†  3.22 ± 0.1*†  46.8 ± 2*†  23.3 ± 1* | 4.1 ± 0.1*†  3.27 ± 0.1*†  43.2 ± 2*†  21.1 ± 1*† |
| CsA-NP at 1.0 mg/kg | LVEDD, mm  LVEDS, mm  EF, %  FS, % | 3.59 ± 0.1  2.50 ± 0.1  59 ± 1  30 ± 1 | 3.95 ± 0.1*†  2.9 ± 0.1*†  53 ± 3†  27 ± 2† | 4.0 ± 0.1*†  3.0 ± 0.1*†  50 ± 2*†  25 ± 1† | 4.1 ± 0.1*†  3.1 ± 0.1*†  49 ± 2*†  25 ±1*† |

The data represent the mean ± SEM (N=10 each).

*P<0.05 versus baseline and †P*<*0.05 versus the saline group compared using a one-way ANOVA followed by Bonferroni’s multiple comparison test.

**Supplemental Table 5**. The effects of cyclosporine A (CsA) and nanoparticles incorporated with CsA (CsA-NP) on blood pressure and heart rate.

|  | | **Weeks after IR** | | | |
| --- | --- | --- | --- | --- | --- |
| **Groups** | | **Baseline** | **1 week** | **2 weeks** | **4 weeks** |
| Saline | Blood pressure, mmHg  Systolic  Mean  Heart rate | 109 ± 3  74 ± 3  677 ± 9 | 98 ± 1  68 ± 2  643±12 | 107 ± 2  71 ± 2  670±21 | 107 ± 3  72 ± 3  686±13 |
| CsA at 1.0 mg/kg | Blood pressure, mmHg  Systolic  Mean  Heart rate, bpm | 102 ± 2  75 ± 1  695 ± 6 | 99 ± 2  64 ± 4  650 ± 17 | 105 ± 2  69 ± 4  668 ± 13 | 109 ± 3  70 ± 4  661 ± 12 |
| CsA at 10 mg/kg | Blood pressure, mmHg  Systolic  Mean  Heart rate, bpm | 101 ±1  67 ± 3  665 ± 15 | 106 ± 2  63 ± 4  684 ± 8 | 110 ± 2  70 ± 3  673 ± 7 | 105 ± 4  64 ± 3  693 ± 12 |
| CsA-NP at 1.0 mg/kg | Blood pressure, mmHg  Systolic  Mean  Heart rate, bpm | 105 ± 3  76 ± 2  681 ± 8 | 105 ± 2  70 ± 2  619 ± 20 | 110 ± 2  76 ± 3  688 ± 10 | 110 ± 3  74 ± 4  667 ± 12 |

The data represent the mean ± SEM (N=10 each). The data were compared using a one-way ANOVA followed by Bonferroni’s multiple comparison test.

**Supplemental Table 6.** Nanoparticle size of CsA-NP and FITC-NP.

|  | dynamic light scattering | | transmission electron microscope | scanning electron microscope | |
| --- | --- | --- | --- | --- | --- |
|  | Nanoparticle Size (nm) |  | Nanoparticle Size (nm) |  | Nanoparticle Size (nm) |
| CsA-NP  FITC-NP | 174 ± 6  222 ± 5 |  | 60 ± 15  91 ± 22 |  | 101 ± 26  164 ± 42 |

The data represent the mean ± SD (n=3).

**Supplemental References**

1 Katsuki, S. *et al.* Nanoparticle-mediated delivery of pitavastatin inhibits atherosclerotic plaque destabilization/rupture in mice by regulating the recruitment of inflammatory monocytes. *Circulation* **129**, 896-906, doi:10.1161/CIRCULATIONAHA.113.002870 (2014).

2 Vasquez, K. O., Casavant, C. & Peterson, J. D. Quantitative whole body biodistribution of fluorescent-labeled agents by non-invasive tomographic imaging. *PloS One* **6**, e20594, doi:10.1371/journal.pone.0020594 (2011).

3 Molica, F. *et al.* Cannabinoid receptor CB2 protects against balloon-induced neointima formation. *Am J Physiol Heart Circ Physiol* **302**, H1064-1074, doi:10.1152/ajpheart.00444.2011 (2012).

4 Gomez, L., Paillard, M., Thibault, H., Derumeaux, G. & Ovize, M. Inhibition of GSK3beta by postconditioning is required to prevent opening of the mitochondrial permeability transition pore during reperfusion. *Circulation* **117**, 2761-2768, doi:10.1161/CIRCULATIONAHA.107.755066 (2008).

5 Boengler, K., Hilfiker-Kleiner, D., Heusch, G. & Schulz, R. Inhibition of permeability transition pore opening by mitochondrial STAT3 and its role in myocardial ischemia/reperfusion. *Basic Res Cardiol* **105**, 771-785, doi:10.1007/s00395-010-0124-1 (2010).

6 Lim, S. Y., Davidson, S. M., Hausenloy, D. J. & Yellon, D. M. Preconditioning and postconditioning: the essential role of the mitochondrial permeability transition pore. ***Cardiovasc Res*** **75**, 530-535, doi:10.1016/j.cardiores.2007.04.022 (2007).

7 Dow, J. & Kloner, R. A. Postconditioning does not reduce myocardial infarct size in an in vivo regional ischemia rodent model. *J Cardiovasc Pharmacol Ther* **12**, 153-163, doi:10.1177/1074248407300897 (2007).

8 Huhn, R. *et al.* Hyperglycaemia blocks sevoflurane-induced postconditioning in the rat heart in vivo: cardioprotection can be restored by blocking the mitochondrial permeability transition pore. *Br J Anaesth* **100**, 465-471, doi:10.1093/bja/aen022 (2008).

9 Liu, L., Zhu, J., Brink, P. R., Glass, P. S. & Rebecchi, M. J. Age-associated differences in the inhibition of mitochondrial permeability transition pore opening by cyclosporine A. *Acta Anaesthesiol Scand* **55**, 622-630, doi:10.1111/j.1399-6576.2011.02421.x (2011).

10 Argaud, L. *et al.* Specific inhibition of the mitochondrial permeability transition prevents lethal reperfusion injury. *J Mol Cell Cardiol* **38**, 367-374, doi:10.1016/j.yjmcc.2004.12.001 (2005).

11 Pagel, P. S. & Krolikowski, J. G. Transient metabolic alkalosis during early reperfusion abolishes helium preconditioning against myocardial infarction: restoration of cardioprotection by cyclosporin A in rabbits. *Anesth Analg* **108**, 1076-1082, doi:10.1213/ane.0b013e318193e934 (2009).

12 Krolikowski, J. G. *et al.* Inhibition of mitochondrial permeability transition enhances isoflurane-induced cardioprotection during early reperfusion: the role of mitochondrial KATP channels. *Anesth Analg* **101**, 1590-1596, doi:10.1213/01.ANE.0000181288.13549.28 (2005).

13 Karlsson, L. O., Bergh, N. & Grip, L. Cyclosporine A, 2.5 mg/kg, Does Not Reduce Myocardial Infarct Size in a Porcine Model of Ischemia and Reperfusion. *J Cardiovasc Pharmacol Ther*, doi:10.1177/1074248411407636 (2011).

14 Karlsson, L. O. *et al.* Cyclosporine does not reduce myocardial infarct size in a porcine ischemia-reperfusion model. *J Cardiovasc Pharmacol Ther* **15**, 182-189, doi:10.1177/1074248410362074 (2010).

15 Lie, R. H. *et al.* Post-conditioning with cyclosporine A fails to reduce the infarct size in an in vivo porcine model. *Acta Anaesthesiol Scand* **54**, 804-813, doi:10.1111/j.1399-6576.2010.02241.x (2010).

16 Skyschally, A. & Heusch, G. Reduction of myocardial infarct size by dronedarone in pigs--a pleiotropic action? ***Cardiovasc Drugs Ther*** **25**, 197-201, doi:10.1007/s10557-011-6300-1 (2011).

17 Piot, C. *et al.* Effect of cyclosporine on reperfusion injury in acute myocardial infarction. *N Engl J Med* **359**, 473-481, doi:10.1056/NEJMoa071142 (2008).

18 Ghaffari, S., Kazemi, B., Toluey, M. & Sepehrvand, N. The Effect of Pre-thrombolytic Cyclosporine-A Injection on Clinical Outcome of Acute Anterior ST-Elevation Myocardial Infarction. *Cardiovasc Ther*, doi:10.1111/1755-5922.12010 (2012).

19 Cung, T. T. *et al.* Cyclosporine before PCI in Patients with Acute Myocardial Infarction. *N Engl J Med* **373**, 1021-1031, doi:10.1056/NEJMoa1505489 (2015).
